# Special Issue “C-Reactive Protein and Cardiovascular Disease: Clinical Aspects”

**DOI:** 10.3390/jcm11133610

**Published:** 2022-06-22

**Authors:** Ahmed Sheriff

**Affiliations:** 1 Division of Gastroenterology, Infectiology and Rheumatology, Medical Department, Charité University Medicine, 12200 Berlin, Germany; ahmed.sheriff@charite.de; 2 Pentracor GmbH, 16761 Hennigsdorf, Germany

## 1. Prologue: The Prehistoric Antibody CRP and Its Immune Complexes

This Special Issue focuses on the clinical relevance of C-reactive protein. Most physicians are familiar with it as a diagnostic biomarker. Only a few have realised that it can be a pathomolecule. After all, biomarker is of course not a physiological function. The main task of CRP is to mark cells to be disposed of, which has been shown for decades in various animal models and has been broken down to the molecular detail [1,2]. For several decades, CRP has been established as an extremely sensitive, reliable and early indicator of inflammatory and tissue-destructive processes. Following an acute phase stimulus, up to 1000-fold increased values can be measured.

This prototype of the human acute phase protein has been considered an inflammatory marker since it was first described by Tillet and Francis in 1930 [3].

However, the mere use of CRP as a readily measurable inflammation marker neglects the biological function of the protein.

CRP is a serum protein and a mediator of innate immunity. The diverse functions of CRP across all living species led to the conclusion that CRP is a prehistoric precursor of all antibodies in the evolutionarily much later appearing mammals.

Already in the horseshoe crab (Limulus), a “living fossil” at least 250 million years old, CRP forms immune complexes together with complement and thus assumes defence functions. At the same time, it acts phylogenetically as an antibody in numerous species, such as fish, which have no adaptive immune system. In humans, its functions are complex and part of re-intensified research.

It is now accepted, even if not everyone is aware of it, that CRP plays a central role in the development of inflammation-related tissue damage [4]. CRP activates (like antibodies) the complement system via the classical pathway [5] and macrophages via Fc receptors [6,7]. Therefore, CRP, like antibodies, binds to Fc receptors.

## 2. The CRP Increase of the First 48 h

Most recently, a significant correlation of the CRP increase after myocardial infarction with the size of the damage was also shown in humans, as well as the reduction of the damage by CRP removal [2,8]. The relevance of the initial increase in CRP levels in the first approximately 48h for prognosis is described by three articles in this Special Issue [9,10,11]. This is confirmed by several recent articles [8,12]. This is an additional clear indication that attention should be paid to CRP in terms of pathological properties.

## 3. CRP Triggers the Disposal of Hypoxic and Ischaemic Cells

The findings from the removal of CRP after STEMI or in severe COVID-19 by CRP apheresis are summarised in the article by Torzewski et al. [13]. The influence of CRP in other cardiovascular disease patterns (atherosclerosis, myocarditis and dilated cardiomyopathy, stroke) and autoimmune diseases (e.g., rheumatoid arthritis, ulcerative colitis, Crohn’s disease, psoriasis, giant cell arteritis) is also discussed. It is also reported here which other drugs are/were already tried to block CRP or reduce its amount.

Very impressively, it was shown by Esposito et al. [14] how beneficial the removal of CRP is in severe COVID-19. The treatments were performed within the CACOV registry to allow for scientific evaluation. The impressive before/after CT and X-ray images of the patients’ lungs speak volumes. This is a very exciting report on a poorly known treatment option for severe COVID-19. Even though it is not a clinical trial, the results are remarkable. Not only was the mortality rate very low in a patient group where >40% would have been expected, but the normally progressive damage to the lungs was also reversed. Additionally, the 50% mortality rate within 12 months of hospitalisation was not seen in this severe cohort. A case series from the same registry (CACOV) with almost the same outcome was recently reported from another hospital [15]. The report by Esposito et al. spectacularly confirms that oxygen-deprived cells get the problem of being disposed of because of CRP, and this actually does not happen when a sufficient amount of CRP is removed from the blood plasma.

The inflammation in the lungs and other tissues caused by the severe acute respiratory syndrome coronavirus 2 (SARS-CoV-2) was and is a disease with a high mortality rate, for which there were still no comprehensive, effective and approved treatment guidelines in spring 2021, but only recommendations.

Even the first publications from Wuhan and later from Italy and the USA showed that this is not an infectious disease in the classical sense, but a strongly immunologically controlled disease [16,17,18]. Accordingly, a deeper understanding of the specific inflammatory process is essential.

Systemic inflammation, measurable for example by C-reactive protein, is correlated with thromboembolic events, acute renal failure, severe courses, ventilation requirement and intensive care requirement, as well as high mortality and high post-discharge mortality in COVID-19 [19,20,21].

This increased plasma CRP level correlates inversely with prognosis in all publications published since then. This is scientific consensus. The odds ratio for mortality increases with the amount of CRP and rises dramatically to over 23 at CRP > 250 mg/L [22].

## 4. CRP and Heart Failure

Zaczkiewicz et al. [23] present an interesting article reporting data from a prospective observational study at a single centre—60 patients with decompensated heart failure. The aim of this study was to investigate whether CRP plasma levels could be reduced by digoxin in addition to optimal medical treatment in patients with heart failure and reduced left ventricular ejection fraction who were hospitalised with decompensated heart failure (NYHA class III and IV). The authors investigated an important issue in the heart failure population. Due to standard heart failure treatment, CRP levels were significantly lower in each group at day 21–day 1 and day 21–day 3. Comparison of the extent of CRP plasma level reduction at day 21–day 3 between the two groups showed borderline significance (*p* = 0.051). Despite the fact that statistical significance was not reached between the groups studied and the number of study participants is small, the study will add to the current knowledge in this field. This study can serve as a basis for further research.

## 5. CRP and Arterial Stiffness Are Associated with the Development of Cardiovascular Disease

Using data from a retrospective single-centre study, Kim and colleagues showed that elevated levels of brachial-ankle pulse wave velocity (baPWV) and C-reactive protein (CRP) were associated with serious adverse cardiovascular events (MACE), even after adjustment for covariates. Furthermore, they showed that the combination of baPWV and CRP further stratified subjects’ risk. They concluded that the combination of CRP and baPWV was better at predicting future cardiovascular death than either value alone. The concepts of the study are easy to understand, and the combination of baPWV and CRP in this study has some innovations [24].

## 6. CRP in Systemic Lupus Erythematosus

Two articles address systemic lupus erythematosus (SLE). Both describe the cardiovascular risk as a function of the CRP concentration. This sounds a bit paradoxical, as SLE patients are characterised by their inability to synthesise high enough amounts of CRP. Allow the authors to illustrate this point for you. The review by Enocsson et al. [25] summarizes the biological effect of CRP in autoimmune conditions and additionally highlights the role of CRP for cardiovascular diseases. It also takes into account the influence of other inflammatory and/or clearance related proteins such as IL6, interferon, complement or CRP autoantigens. Pesqueda-Cendejas and colleagues [26] report that high CRP levels were connected with high cardiometabolic risk and high clinical disease activity in SLE patients. This implication is not a surprise; however, the cohorts are very well selected and consist of a large number of female patients with the same ethnicity.

## 7. CRP’s Influence on G-Protein Coupled Receptors

In another article [27], a new, previously undescribed property of CRP is reported. CRP interferes with the desensitization of G-protein coupled receptors (GPCRs) and has to be considered as a novel regulator of adrenergic, angiotensin-1 and endothelin receptors. This is a surprise because CRP’s molecular action has so far only been investigated on, e.g., Fc receptor γRII (FcγRII) and in the context of macrophage activation and its role as an archaic antibody-like molecule. In addition, CRP induces the classical complement pathway after binding to its ligand, which produces immune complexes. However, the direct influence of CRP on the cardiovascular system of rabbits has also been reported recently [28], which has nothing to do with a lack of oxygen supply. Surprisingly, the effect takes place on well energised cells. What might this mean with regard to tachycardia in high inflammation?

## 8. Epilogue

The articles presented in this Special Issue reveal a broad spectrum of indications with CRP involvement. The reports unanimously support the view that CRP has a dark side. In addition to this new perspective about pathological properties of CRP, two other new aspects are crystallising. One is that the initial rate of CRP synthesis in an acute illness such as sepsis or myocardial infarction allows an excellent prognosis in terms of mortality or cardiac function/scar area. The other is the surprising finding that CRP impairs the desensitisation of GPCRs without the need for any further damaging process. I am curious to learn what other features CRP has kept from us so far.
